# Are Married Men Healthier than Single Women? A Gender Comparison of the Health Effects of Marriage and Marital Satisfaction in East Asia

**DOI:** 10.1371/journal.pone.0134260

**Published:** 2015-07-31

**Authors:** Woojin Chung, Roeul Kim

**Affiliations:** 1 Department of Health Policy and Management, Graduate School of Public Health, Yonsei University, Seoul, Korea; 2 Institute of Health Services Research, Yonsei University, Seoul, Korea; 3 National Tobacco Control Center, Korea Health Promotion Foundation, Seoul, Korea; Örebro University, SWEDEN

## Abstract

**Background:**

Although Asian societies are remarkably different from Western societies in terms of sociocultural characteristics, little is known about the gender differences in the health effects of marriage and marital satisfaction in Asian countries.

**Methodology/Principal Findings:**

Using a randomly sampled dataset from the 2006 East Asian Social Survey comprising 8528 individuals from China, Japan, Taiwan, and South Korea, this study performs analyses using a multivariate logistic regression model to predict the probability for a man or a woman to report poor health. Our results differ quite significantly from those of most studies focusing on Western countries. Considering marital satisfaction, there may be no health benefits from marriage for a specific gender in a given country, because the health loss associated with a dissatisfied marriage usually supersedes the health benefits from marriage. Moreover, women may reap greater health benefits from marriage than men. Additionally, those most likely to report poor health are found to be married and dissatisfied men or women, rather than never-married individuals.

**Conclusion/Significance:**

The present study argues the need to design and carry out a gender- and country-specific social health policy approach to target individuals suffering from poor health, thereby reducing the gender differences in health status.

## Introduction

There is ample evidence to suggest that marriage provides health benefits; married individuals display better physical and mental health than their single counterparts [1,2]. Focusing on the gender differences in the effects of marriage on individual health, some studies in particular have shown that marriage might benefit men’s health more significantly than women’s [3,4]. However, other studies have found that marriage provided equal health benefits to men and women [1,2].

Furthermore, focusing on married people, some studies have shown that marital satisfaction was important for the health of married individuals [5,6]. As regards the gender differences in terms of marital satisfaction and health in married individuals, other studies have found that marriage satisfaction had a greater positive impact on women’s health than men’s [7,8]. By contrast, other studies have reported no gender differences in the association between marital satisfaction and health.

Despite the well-known gender discriminations in East Asia [9,10], most studies investigating the gender differences in the effects of marriage and marital satisfaction on individual health have focused on Western societies. In the present study therefore, we aimed to explore the gender differences in the health benefits of marriage, as well as in the association between marriage, marital satisfaction and individual health, in East Asia.

The main thrust of the present study was the hypothesis that marriage benefits not only the health of men, but also that of women. Flowing from this, we posited a subordinate hypothesis that the individuals with the highest risk of poor health were those who had never married, irrespective of gender. Additionally, we hypothesized that among those experiencing positive health benefits from marriage, men were likely to enjoy greater benefits than women.

Data were drawn from the 2006 East Asian Social Survey, which consisted of individuals in China, Japan, Taiwan, and South Korea (hereafter, Korea). For each gender in each country, the difference in self-rated health was compared among the different groups of individuals categorized according to their marital satistaction and status. Multivariate logistic regression models were employed to predict the probability that a man or a woman would have poor self-rated health if he or she belonged to a specific group for marriage and marital satisfaction and met the mean values for other socio-demographic characteristics.

## Gender Differences in Marriage, Marital Satisfaction, and Health Outcomes

A review of the Western literature on marriage and health reveals that marriage is positively associated with individual health in both genders; married people are shown to have better physical and mental health than their unmarried counterparts [1–4,11,12]. Analyzing 11,112 individuals covered by the Panel Study of Income Dynamics in the U.S., Lillard and Waite [12] found that both married women and men showed a substantially lower risk of dying than unmarried participants. Lindstrom [2] analyzed 27,757 individuals aged 18 to 80 using data from a 2004 public health survey in Sweden and found that, for both women and men, never-married individuals and divorced individuals had a significantly higher odds ratio of poor self-rated health than married or cohabiting individuals. Law and Sbarra [1] analyzed 791 older adults, who were interviewed at three different time points over an eight-year period, between 1992 and 2000, in Australia. They found that being married was associated with a lower level of depressed mood both in men and in women. Regarding gender differences in the health benefits of marriage, meanwhile, some studies suggested that marriage might benefit men’s health more than women’s [3,4,11]. Umberson [3], who analyzed national panel surveys conducted in the U.S. in 1986 and 1989, showed that men reaped greater health benefits from marriage than women, partly because women do more to promote their spouse’s health.

Highlighting the association between marital satisfaction and health in married individuals, other studies have shown a significant association between the two [7,8,13–16]. Among these studies, some reported no gender difference in the association between marital satisfaction and health [13,17]. Wickrama et al. [13] analyzed a dataset of married individuals comprising 367 women and 340 men in the U.S. and examined the influence of stressful marital and parental relationships on the hazard rates and survival probabilities for hypertension. Their results showed that marital stress influenced the health of both wives and husbands equally. They assumed that these findings were attributable to the relatively stable marriage statuses of the wives and husbands included in their sample, as the spouses they surveyed had been married for an average of more than two decades. Analyzing 2,348 individuals from the American Changing Lives panel survey in the U.S., Williams [16] found that marriage dissatisfaction undermined the psychological well-being of both women and men to a similar extent. In that study, the psychological well-being factors included depression and life satisfaction. Although the measurements presented limitations that made it difficult to generalize the results, the author suggested that the absence of gender differences might reflect the recent improvement in women’s status and economic opportunities in the U.S.

On the other hand, other studies have revealed significant gender differences in the relationship between marital satisfaction and health [7,8,15]. Levenson et al. [7] studied 156 U.S. couples covering two generations–one group in middle age (40–50 years) and the other on the threshold of old age (60–70 years). They found that in both groups, the relationship between marital satisfaction and health was stronger in women than in men. In satisfied marriages, the health of the wives and husbands was equivalent. In dissatisfied marriages however, the wives reported more mental and physical health problems than their husbands. Levenson *et al*. explained that the responsibility for confronting marital conflict and attempting to heal an ailing marriage fell primarily on the wives, and that the cost they had to bear in taking on the emotional and physical work associated with this responsibility depleted their mental and physical health reserves. St John and Montgomery [15] also reported some gender differences in the health effects of marriage and marital satisfaction. Examining 1,751 Canadian adults aged 65 years and older between 1991~1992, they found that with reference to the health of unmarried individuals, marital satisfaction only improved the health of married men, and marital dissatisfaction only affected the health of married women.

Meanwhile, no study of Asian countries to date has used a nationwide, randomly sampled dataset including both unmarried and married individuals to explore the gender differences in the health effects of marriage and marital satisfaction. The few existing studies of East Asian countries have fallen into two categories: examinations of the gender differences in the relationship between marital status and health [18–21], or between marriage quality and health in married people [22]. Shek [22] analyzed 738 married men and 761 married women in Hong Kong and found that the impact of marriage quality on individuals’ well-being was generally greater in women than in men. In his study, the husbands generally reported a greater level of marital satisfaction than their wives.

According to related studies, the experiences of marriage and family culture in East Asian and Western countries are somewhat different. For example, marriage in Asia remains a nearly universal phenomenon, and divorce continues to be stigmatized [23]. In addition, the strong emphasis on familism in China means that many marriage-related decisions are still influenced by parental interventions [23–25]. Pimentel [23] examined marital satisfaction in 1,778 couples from urban China and found that the parental approval of mates had a strong influence on the marriage quality. Other Asian countries such as Japan and Korea also display strong commonalities in their kinship systems, which remain patrilineal [10,26,27]. Exploring the relationship between women’s education and marriage using data from the Japanese National Fertility Surveys, Raymo and Iwasawa [26] found that as Japan remains a traditional society with a high rate of gender inequality–unlike most Western, industrialized societies–, the marriage rates of highly educated women had been declining. From a cross-cultural perspective, the lack of related studies in Asia motivates one to ask whether any gender differences exist in the health effects of marriage and marital satisfaction, and whether such differences differ from those observed in Western societies. A cross-country analysis under a coordinated research setting would therefore be helpful to determine whether evidence obtained from a country can be valid in another country.

## Methods

### Data Source and Study Sample

We used data from the 2006 East Asian Social Survey (hereafter, EASS) to test the above-mentioned research hypotheses. The EASS is the East Asian version of the European Social Survey, which has been administrated in 30 European countries for several decades. For the EASS, China, Japan, Taiwan, and Korea share a common module via a General Social Survey-type questionnaire, and each country additionally implements a nationally representative sample survey through a multistage stratified random sampling method. The EASS provides details of the survey methodology in an online report [28].

We chose the 2006 EASS dataset because it was the only nationwide dataset containing information on self-rated health, marriage, marital satisfaction, as well as other individual socio-demographic characteristics, and allowing us to compare different countries in East Asia. The dataset drew on face-to-face interviews conducted in each country between June and December 2006. Out of a total of 8,842 participants aged 20 years and older, we excluded 314 individuals (3.55%) who had not responded to the questionnaire items needed in the study. 8,528 respondents–consisting of 3,051 individuals in China, 1,975 in Japan, 1,981 in Taiwan, and 1,521 in Korea–were therefore included in our final sample.

The EASS data archive provides publicly available data from the participants, whose identities are undisclosed. Due to the limited timeframe for the survey interviews, verbal informed consent was obtained from all participants, and written consent waivers were given by the ethics committee. Ethical approval for this study was granted by the review board of the institution where the authors were working.

### Variables

We measured marital satisfaction based on the married participants’ responses to the relevant item in the interview questionnaire. During the survey, married individuals were asked, “All things considered, how would you describe your marriage? Would you say that you are very satisfied or dissatisfied with your marriage?’ and were encouraged to answer on a 5-point scale. We grouped the answers into three categories: (a) married and satisfied, (b) married and neutral, and (c) married and dissatisfied. Next, in order to construct the marriage and marital satisfaction variable, we added these three groups of married individuals to the remaining two marital status groups: (a) married and satisfied, (b) married and neutral, (c) married and dissatisfied, (d) formerly married, and (e) never married.

The question asking participants to rate their own health was: “How would you rate your own health?”. To rate their health at the time of the interview, the participants were given five options: very good, good, fair, bad, and very bad. A dichotomous outcome variable was constructed with a value of 1 (poor health) for “bad” or “very bad” and 0 (better health) otherwise.

Various socio-demographic characteristics were included as potential covariates: namely, the gender, age (20−29, 30−39, 40−49, 50−59, and 60 years and above), educational attainment, employment status, self-rated social class, and religion. The highest education level was used as the indicator for educational attainment, which was divided into four categories: lowest (no or lowest qualification), low (higher secondary completed), high (above higher secondary), and highest (university degree). According to their employment status, the individuals were categorized into two groups: not-employed (the unemployed, the retired, the disabled permanently out of the labor force, students, and housewives), and employed. ‘Top-bottom self-placement’ on a 10-point scale is used for the self-rated social class in Taiwan, Japan, and Korea and ‘Class identification’ on a 5-point scale is available in China. The question involving ‘Top-bottom self-placement’ was: ‘In our society there are groups that tend to be towards the top and groups that tend to be towards the bottom. Below is a scale that runs from bottom to top. Where would you put yourself on this scale?’ Available choices are numerical on a 10-point scale from 1 (lowest) to 10 (highest). The question regarding ‘Class identification’ was: ‘In your opinion, which level do you and your family belong in terms of your personal and family socio-economic status? (Choose one in each column)’. Choices are 1 (upper level), 2 (upper middle level), 3 (Middle level), 4 (lower middle level), and 5 (lower level). We used the responses to the question about ‘Your socio-economic status’ rather than ‘Your family’s socio-economic status’ as the class identification variable for China. We converted the 10-point scale of ‘Top-bottom self-placement’ into a 5-point scale and merged the two highest class identification categories because of the smaller number of applicable cases in these groups. Moreover, we reversed the ‘Class identification’ scale and merged the two highest categories. As a result, we obtained four class identification index levels (lowest, low, high, and highest) for analysisThe participants were also categorized on the basis of their religious affiliation. Considering the distribution of the responses as to religion, we dichomized the responses as Buddhist and “others”.

### Statistical Analyses

A five-step analysis was performed for each country. First, the baseline characteristics of the sample individuals for each gender and country group were examined. Second, the individual characteristics by country group were compared between men and women using χ^2^ tests. Third, the differences in the proportion of individuals reporting poor health were examined for each categorical characteristic according to the gender and country group, using χ^2^ tests. Fourth, because the gender modified the effect of marriage and marital satisfaction on the report of poor health in the preliminary analysis, two multivariate logistic analyses were performed for each country group according to the two main effect terms of the gender and the marriage and marital satisfaction, as well as with one relevant interaction term: Model 1 included the main effects of the two variables and their interaction effect; and Model 2 included all remaining covariates in addition to the variables in Model 1. Lastly, after obtaining fully adjusted ORs, we obtained a prediction of the probability, with 95% confidence intervals (95% CI), that an individual would have poor self-rated health if he or she belonged to a certain marriage and marital satisfaction group and met the mean values for other socio-demographic characteristics of the country group.

The adjusted odds ratios (OR) were presented with 95% CIs and the significance was set at an alpha level of 0.05. Likelihood ratio tests were performed to find the model with the best fit. C-statistics and a Hosmer-Lemeshow goodness-of-fit test were performed to test how well the logistic analysis fit the actual data. The data analyses were performed using SAS, version 9.2 (SAS Institute Inc., Cary, NC, USA) and STATA, version 13 (StataCorp, College Station, TX, USA).

## Results

The characteristics of the sample individuals by gender and country group are presented in Table 1. A higher proportion of women than men reported poor health (bad or very bad) in all countries, with the highest value found in Korean women (22.45%). For those in dissatisfied marriages, the proportion of women reporting poor health was higher than that of men, with the highest incidence in Japanese women (9.48%).

**Table 1:** Characteristics of sample individuals by gender and country, the 2006 East Asian Social Survey.

Characteristics	China					Japan					Taiwan					South Korea				
	Men		Women			Men		Women			Men		Women			Men		Women		
	No.	(%)	No.	(%)	*p*	No.	(%)	No.	(%)	*p*	No.	(%)	No.	(%)	*p*	No.	(%)	No.	(%)	*p*
Total	1378	(100.00)	1673	(100.00)		889	(100.00)	1086	(100.00)		1003	(100.00)	978	(100.00)		688	(100.00)	833	(100.00)	
Poor self-rated health	157	(11.39)	286	(17.10)	< .001	110	(12.37)	135	(12.43)	0.969	134	(13.36)	187	(19.12)	< .001	92	(13.37)	187	(22.45)	< .001
Marriage and marital satisfaction					< .001					< .001					< .001					< .001
Married and neutral	127	(9.22)	228	(13.63)		171	(19.24)	261	(24.03)		71	(7.08)	77	(7.87)		112	(16.28)	223	(26.77)	
Married and satisfied	959	(69.59)	1142	(68.26)		474	(53.31)	386	(35.55)		548	(54.64)	487	(49.81)		322	(46.80)	287	(34.45)	
Married and dissatisfied	34	(2.47)	55	(3.29)		20	(2.25)	103	(9.48)		21	(2.09)	42	(4.29)		30	(4.36)	64	(7.68)	
Formerly married	66	(4.79)	118	(7.05)		68	(7.65)	188	(17.31)		63	(6.28)	152	(15.54)		35	(5.09)	122	(14.65)	
Never married	192	(13.93)	130	(7.77)		156	(17.55)	148	(13.63)		300	(29.91)	220	(22.49)		189	(27.47)	137	(16.45)	
Age, years					0.580					0.088					< .001					0.179
≤29	248	(18.00)	306	(18.29)		96	(10.80)	121	(11.14)		235	(23.43)	177	(18.10)		138	(20.06)	139	(16.69)	
30–39	326	(23.65)	420	(25.11)		153	(17.21)	163	(15.01)		165	(16.45)	227	(23.22)		155	(22.53)	227	(27.25)	
40–49	318	(23.08)	404	(24.15)		122	(13.72)	188	(17.31)		227	(22.63)	211	(21.57)		194	(28.19)	219	(26.28)	
50–59	304	(22.06)	336	(20.08)		170	(19.12)	228	(20.99)		166	(16.55)	180	(18.40)		87	(12.65)	105	(12.61)	
≥60	182	(13.21)	207	(12.37)		348	(39.15)	386	(35.55)		210	(20.94)	183	(18.71)		114	(16.57)	143	(17.17)	
Educational attainment					< .001					< .001					0.089					< .001
Lowest	797	(57.83)	1118	(66.82)		165	(18.56)	199	(18.32)		371	(36.99)	415	(42.43)		117	(17.01)	219	(26.29)	
Low	341	(24.75)	364	(21.76)		402	(45.22)	580	(53.41)		260	(25.92)	225	(23.01)		183	(26.60)	278	(33.37)	
High	159	(11.54)	128	(7.65)		44	(4.95)	190	(17.50)		172	(17.15)	162	(16.56)		166	(24.13)	134	(16.09)	
Highest	81	(5.88)	63	(3.77)		278	(31.27)	117	(10.77)		200	(19.94)	176	(18.00)		222	(32.26)	202	(24.25)	
Employed	1305	(94.70)	1446	(86.43)	< .001	650	(73.12)	570	(52.49)	< .001	718	(71.59)	597	(61.04)	< .001	523	(76.02)	395	(47.42)	< .001
Self-rated social class					0.418					0.925					< .001					0.217
Lowest	539	(39.11)	690	(41.24)		66	(7.42)	80	(7.37)		72	(7.18)	46	(4.70)		48	(6.98)	72	(8.64)	
Low	407	(29.54)	500	(29.89)		203	(22.83)	240	(22.10)		164	(16.35)	104	(10.63)		242	(35.17)	255	(30.61)	
High	393	(28.52)	445	(26.60)		475	(53.44)	577	(53.13)		557	(55.53)	593	(60.64)		297	(43.17)	384	(46.10)	
Highest	39	(2.83)	38	(2.27)		145	(16.31)	189	(17.40)		210	(20.94)	235	(24.03)		101	(14.68)	122	(14.65)	
Buddhist	60	(4.35)	124	(7.41)	< .001	248	(27.90)	259	(23.85)	0.041	213	(21.24)	242	(24.74)	0.064	186	(27.03)	242	(29.05)	0.384
Number of individuals	3051					1975					1981					1521				

Note: Formerly married includes widowed, divorced and separated; and p-value is based on the Chi-square test.

In terms of educational attainment, more men than women had reached “the highest level”, and Japanese men demonstrated the highest proportion of highest-level education (31.27%). Chinese women revealed the highest proportion of rating their social class “lowest” (41.24%), which was a sharp contrast to its lowest proportion in Taiwanese women (4.70%). The proportion of Buddhists was the highest in Korean women (29.05%). Most characteristics showed significant gender differences in their distribution.

Table 2 illustrates the way the two variables of (1) marriage and marital satisfaction and (2) gender were associated with the report of poor health in each country. In Model 1 (data are not shown due to word limitation), which included the main and interaction effects of the two variables, the interaction effect term was significant in China and Japan. Meanwhile, in Model 2, which incorporated all the remaining covariates in addition to the variables in Model 1, at least one of the interaction effect terms of the two variables was significant in Japan and in Korea, and was also very close to the significance level in China (*p* = 0.056) and in Taiwan (*p* = 0.055). No significant lack of fit was found for the models through either the C-statistics (higher than 0.7 for each country) or the Hosmer-Lemeshow test (*p* > 0.9 for each country). The likelihood ratio tests indicated that Model 2 had a better fit than Model 1 (*p* < 0.001 for each country).

**Table 2:** Fully adjusted associations between two variables (marriage and marital satisfaction; gender) and reported poor health by country, the 2006 East Asian Social Survey.

	China			Japan			Taiwan			South Korea		
	OR	(95% CI)	*p*	OR	(95% CI)	*p*	OR	(95% CI)	*p*	OR	(95% CI)	*p*
Marriage and marital satisfaction												
Married and neutral (MN)	1.00			1.00			1.00			1.00		
Married and satisfied (MS)	0.89	(0.61–1.30)	0.549	0.76	(0.42–1.38)	0.363	1.19	(0.61–2.30)	0.605	0.70	(0.43–1.15)	0.159
Married and dissatisfied (MD)	1.90	(0.94–3.85)	0.074	2.28	(1.17–4.45)	0.015	2.99	(1.19–7.52)	0.020	1.00	(0.51–1.99)	0.989
Formerly married (FM)	1.32	(0.77–2.25)	0.308	2.54	(1.44–4.47)	0.001	1.18	(0.58–2.41)	0.653	1.59	(0.93–2.74)	0.093
Never married (NM)	1.45	(0.56–3.75)	0.443	1.78	(0.84–3.78)	0.134	2.64	(1.17–5.94)	0.019	2.08	(0.95–4.55)	0.068
Gender												
Woman	1.00			1.00			1.00			1.00		
Man	0.40	(0.20–0.81)	0.011	2.08	(1.12–3.86)	0.020	1.33	(0.56–3.15)	0.522	0.41	(0.21–0.82)	0.012
Marriage and marital satisfaction*Man												
MS*Man	1.66	(0.79–3.49)	0.182	0.92	(0.42–2.03)	0.835	0.53	(0.21–1.34)	0.177	1.76	(0.76–4.04)	0.187
MD*Man	0.73	(0.18–3.02)	0.665	0.77	(0.20–2.95)	0.700	0.37	(0.08–1.72)	0.204	5.06	(1.49–17.20)	0.009
FM*Man	1.07	(0.37–3.09)	0.907	0.27	(0.10–0.74)	0.011	0.63	(0.20–1.99)	0.433	1.66	(0.55–4.97)	0.367
NM*Man	3.12	(0.97–10.04)	0.056	0.38	(0.15–0.99)	0.047	0.36	(0.13–1.02)	0.055	1.40	(0.50–3.91)	0.519
Goodness of fit												
C-statistic		0.730			0.710			0.756			0.773	
Hosmer-Lemeshow test			0.941			0.968			0.908			0.991
-2 Log likelihood		2264.42			1357.31			1514.28			1194	
Log likelihood ratio test			< .001			< .001			< .001			< .001
Number of individuals	3051			1975			1981			1521		

Note: Formerly married includes widowed, divorced and separated; OR denotes odds ratio; CI denotes confidence interval; analysis is performed with adjustment for age, education, self-rated social class, employment and religion; and likelihood ratio test was performed relative to the model without the adjustment.

**Fig 1:**
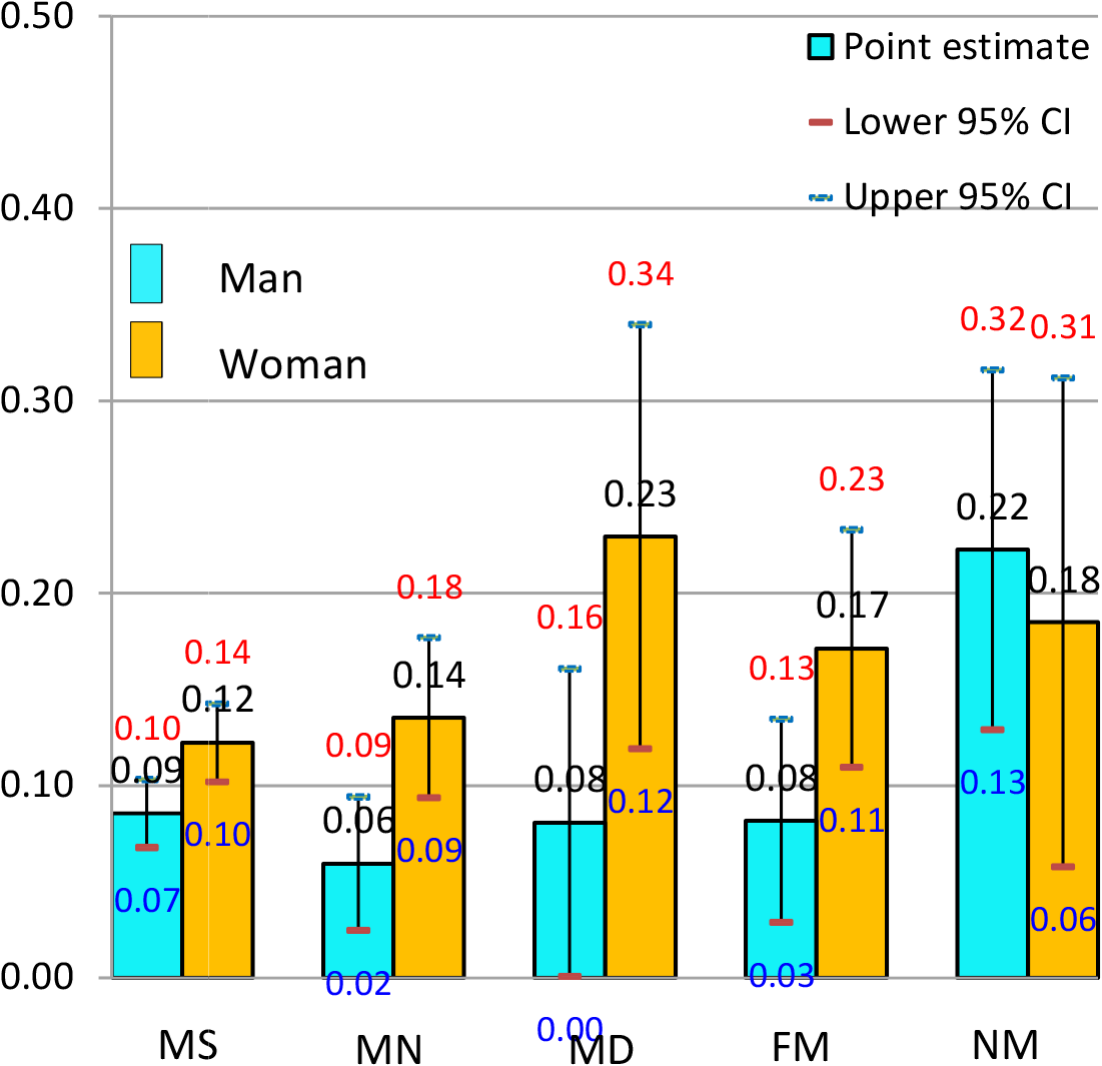
Predicted probability (and 95% confidence intervals) of reporting poor health by gender in China, the 2006 East Asian Social Survey.

Figs 1 through 4 display the predicted probability (hereafter, PREDP) (with 95% CIs) for an individual to report poor health for each category of marriage and marital satisfaction in China, Japan, Taiwan, and Korea, respectively. The predictions were carried out on the basis of the fully adjusted ORs in Model 2, with all the covariates held at their own mean values for each country. In China (Fig 1), men seemed to receive significant health benefits from marriage: the PREDP of poor health reports was lower in any of the three married groups (0.06~0.09) than in the never-married group (0.22). Women also gained health benefits from marriage but not in the married and dissatisfied group; The PREDP of this married and dissatisfied group (0.23) was much higher than that of the never-married group (0.18), implying a potential health loss associated with marriage dissatisfaction. In China therefore, the group with the highest PREDP of reporting poor health was the never-married group for men (0.22), but the married and dissatisfied group for women (0.23). Among the married individuals receiving health benefits from marriage, the drop in PREDP with reference to the never-married group was greater in men (0.13~0.16) than in women (0.04~0.06). In married women, the PREDP of reporting poor health decreased as the marital satisfaction went up.

**Fig 2:**
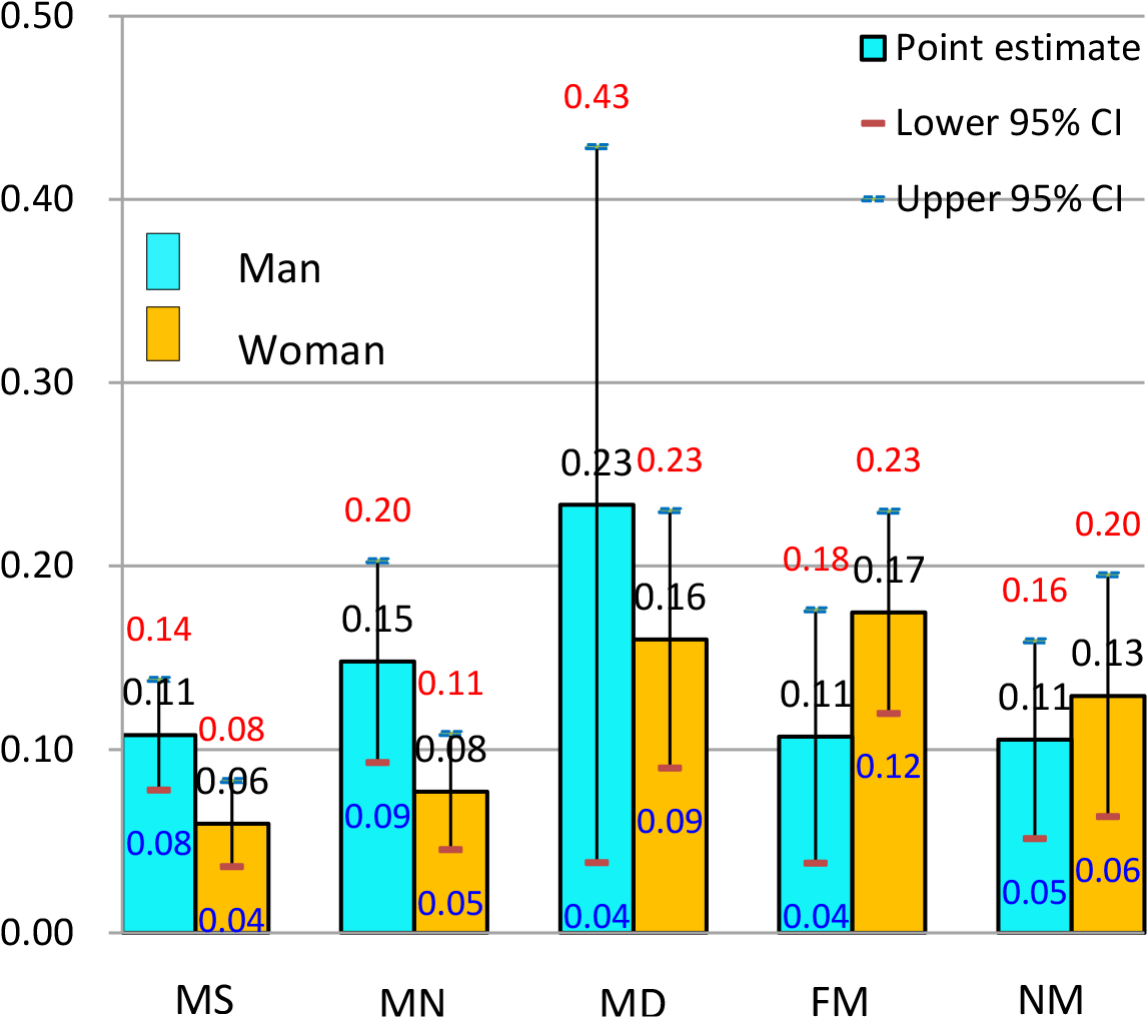
Predicted probability (and 95% confidence intervals) of reporting poor health by gender in Japan, the 2006 East Asian Social Survey.

**Fig 3:**
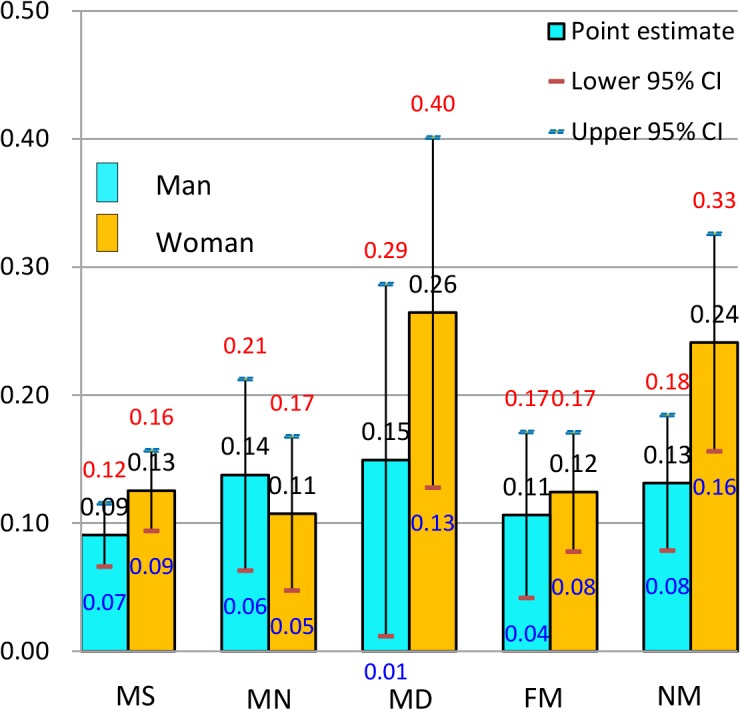
Predicted probability (and 95% confidence intervals) of reporting poor health by gender in Taiwan, the 2006 East Asian Social Survey.

**Fig 4:**
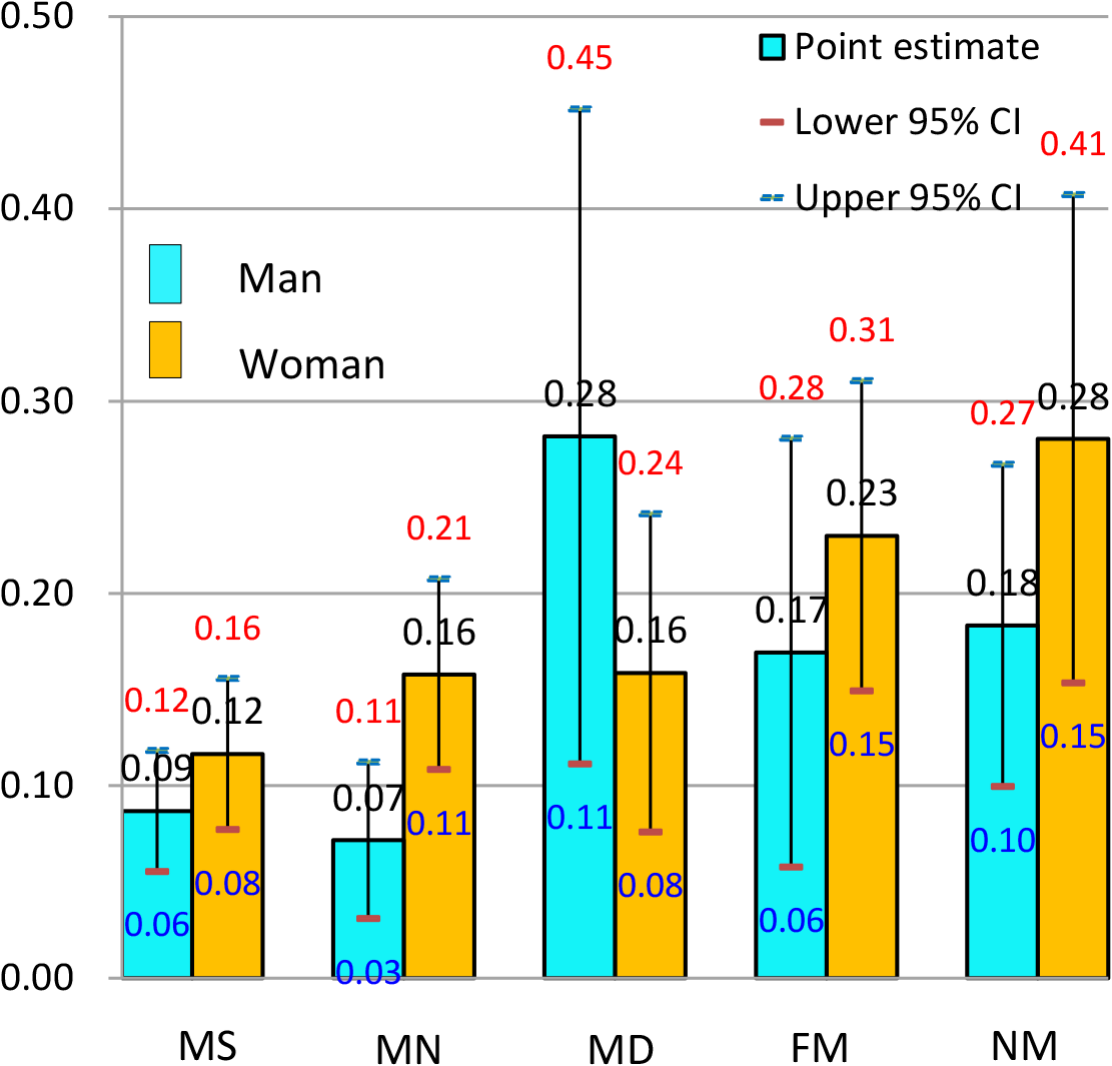
Predicted probability (and 95% confidence intervals) of reporting poor health by gender in South Korea, the 2006 East Asian Social Survey.

In Japan (Fig 2), men seemed to reap no health benefits from marriage: the PREDP of poor health reports from each of the three categories of married men was higher than, or similar to, that of never-married men. The married and dissatisfied group showed the highest PREDP of poor health reporting (0.23), whereas the never-married group revealed the lowest PREDP (0.11), suggesting a sharp decline in health caused by marriage. In women, marriage benefited the health of the married and satisfied and of the married and neutral groups, but the health of the married and dissatisfied group (0.16) was poorer than that of the never-married group (0.13). The highest PREDP of poor health reporting was found in the married and dissatisfied group for men (0.23) and in the formerly married group for women (0.17). Considering the married individuals receiving health benefits from marriage, this benefit was seen to be limited to two groups of married women (those satisfied or neutral with their marriages), with a drop in the PREDP (0.05~0.07). Both men and women showed a monotonically decreasing pattern between the PREDP and marital satisfaction.

In Taiwan (Fig 3), in comparison with never-married men (0.13), married and satisfied men (0.09) seemed to gain health benefits from marriage, whereas married and neutral men (0.14) and those in the married and dissatisfied group (0.15) showed a health loss associated with marriage. In women, in comparison with the never-married group (0.24), some health benefits from marriage were observed in the women who were satisfied (0.13) or neutral (0.11) towards their marriages, but a health loss associated with marriage was observed in the women in dissatisfied marriages (0.26). For both men and women, the group exhibiting the highest PREDP of poor health reporting was the married and dissatisfied group; however, the PREDP in this group was much higher in women (0.26) than in men (0.15). Where the participants benefited in health from their marriages, the magnitude of the health benefit seemed greater in women than in men: a bigger drop from the PREDP for the never-married group was observed in women (0.12~0.13) than in men (0.04). In men, the PREDP seemed to have a negative relationship with marital satisfaction.

Although both men and women received health benefits from marriage in Korea (Fig 4), the married and dissatisfied men group constituted an exception: relative to the never-married men (0.18), the PREDP of the group’s poor health reporting was very high (0.28). The groups presenting the highest PREDP of poor health reports were the married and dissatisfied group for men (0.28) and the never-married group for women (0.28). Among the groups that reaped health benefits from their marriages, the drop in PREDP from the never-married group to the married group was greater in women (0.12~0.16) than in men (0.10~0.11). No monotonic pattern between the PREDP and marital satisfaction was detected either in men or in women.

## Discussion and Conclusions

By analyzing a randomly sampled nationwide dataset including China, Japan, Taiwan, and Korea, the present study suggests that marriage and marital satisfaction are differently associated with individual health according to both gender and country. More specifically, the main findings regarding the study hypotheses can be summarized as follows. First, according to the gender and level of marital satisfaction, marriage may be either positively or negatively associated with self-rated health, and the association between the two factors may vary between the countries: health losses associated with marriage may occur, as evidenced in the three married men groups in Japan; in the married and dissatisfied men group in Korea; and in the married and dissatisfied women group in China, Japan, and Taiwan. Second, the individuals presenting the highest risk of poor health reporting are more likely to be the married and dissatisfied individuals than the never-married or formerly-married, although this likelihood may vary across genders and countries: in men, the highest-risk group was the married and dissatisfied group in Japan, Taiwan, and Korea, but the never-married group in China; in women, the highest-risk group was the married and dissatisfied group in China and Taiwan, the formely married group in Japan, and the never-married group in Korea. Third, depending on the country, among the groups receiving positive health benefits from marriage, women appeared to obtain greater health benefits from marriage than men: this was observed in Japan, Taiwan, and Korea. Lastly, depending on the gender and country, the health of married individuals does not necessarily increase monotonously with their marital satisfaction: this was observed in Chinese, Taiwanese and Korean men, as well as among women in Taiwan and Korea.

In some ways, our findings seem consistent with those of existing studies of Western societies, in that gender differences exist in the association of marriage and marital satisfaction with individual health [3,4,7,8,11,15,29]. Kimmel et al. examined 174 patients undergoing hemodialysis (HD) in the US. Patient recruitment began September 1992 and concluded in March 1996. Their observation period ended in September 1996. They found that women with higher dyadic satisfaction and decreased dyadic conflict were at a decreased mortality risk, but dyadic adjustment indices were associated with differential survival in the larger group of men [8]. However, in other aspects, our findings depart from the above-mentioned studies, as they do not support the theory that men enjoy greater health benefits from marriage than women [3,4,11]. Patten et al. analyzed 36,984 subjects aged ≥ 15 years using the 2002 Canadian Community Health Survey. They found that depression was more prevalent in unmarried men than married men, whereas it was less prevalent in unmarried women than married women [4]. Likewise, we did not observe a stronger relationship between marital satisfaction and health in women than in men [7], or opposite influencing patterns between the genders to the effect that marital satisfaction would increase health only in married men and marital dissatisfaction decrease health only in married women [15].

Further, one distinct finding from the present study is that there might exist a group of individuals suffering health losses from marriage, and the identity of this group may depend on the gender and country, as shown in all of the three married men groups in Japan, in the married and dissatisfied men groups in Taiwan and Korea, and the married and dissatisfied women groups in China, Japan and Korea. This suggests that for a gender in a country, although some benefits to individual health may be derived from the “marriage protection” effects [30,31] and overestimated by the “marital selection” effects [32,33], the health benefits of marriage could be outstripped by the health losses associated with dissatisfied marriages, thereby resulting in a net loss of health.

On the basis of these results, the present study may call for an exploration of the mechanisms by which marriage may work to deteriorate the health of individuals in a variety of familiar and socio-cultural circumstances. Specifically, we may ask the question, “Unlike in the findings of previous Western studies, why is a group of married individuals experiencing health losses because of marriage?” According to our findings, the answer to this question seems to involve at least three dimensional explanations: the gender, the level of marital satisfaction, and the country. However, no study in the public health field has documented the potential answers yet. This may be due both to the absence of clear evidence of gender differences related to these issues in Western societies, and because of the lack of study of these issues in other societies including Asia. Therefore, reviewing the studies broadly relevant to our question, we attempt to dig out potential answers as follows.

First, the marital roles of women and men are different, and the roles of married women tend to be more stressful and demanding, while also less gratifying, than those of married men [11]. Although a married woman may have a job in the labor market, she will nevertheless be expected to take on the traditional responsibilities of caring, nurturing, and kin-keeping–a phenomenon described as the “double burden” [34,35]. Therefore, women’s housework overload and thereby marital dissatisfaction may result in the poor health of married women, varying from country to country. In Asian societies, the husbands are traditionally much less likely to help their wives than in the West, and wives are usually in charge of doing the household chores and raising the children [10,27]. Until recently, this had not changed much in advanced Asian countries. In Japan, married women spend significantly more time doing domestic work than their counterparts in most Western countries [26,36].

Second, marriage has been said to provide emotional benefits such as reducing stress [1,12], whereas unhappy marriages may increase stress [13,17]. Because women seem more likely than men to focus on their emotions, married women are apt to feel more sensitive to emotional changes than married men [29,37], which may cause health deterioration in married and dissatisfied women according to the country.

Third, the responsibility for confronting marital conflict and attempting to heal an ailing marriage often primarily befalls the wife. Husbands, by contrast, tend to buffer themselves from this process by withdrawing from conflictual interactions. Depending on the country, and particularly in East Asia, husbands may view these problems as trivial issues best left for their wives to handle. This withdrawal may function to protect the health of married men, but it may add significantly to the burden placed on the health and emotions of married women [7].

Fourth, the involvement of the parents-in-law in married women’s lives may differ across countries. In Asian countries, which are still patrilineal and son-preferring, after marriage women become the “subordinate daughters” of their mothers-in-law and become responsible for taking care of their husbands’ family members [10,38,39]. A dissatisfying relationship with one’s mother-in-law is an important risk factor for health concerns such as postnatal depression in married women, especially in cases where the woman remains infertile, gives birth to a daughter rather than a son, or women holds different views from her mother-in-law regarding housework [25,40].

Fifth, although the situation may differ slightly between countries, married men tend to be socially forced to work for their wives and children at the expense of their own time and health, as married women are still expected to relinquish their professional careers in order to tend for their husbands and children [26,41]. For example, in Korea, the labour force participation rate of men was 72% in 2012, in contrast to 50% for women, and labourers worked the longest hours on average among OECD member countries [42–44]. Compared to the situation in Western countries, wives in Japan or Korea may push their husbands to work so hard that they may become dissatisfied with their marriage, which may severely deteriorate their physical and mental health and may explain the health loss observed in married and dissatisfied men in these two countries.

Sixth, the freedom to divorce may vary between men and women depending on the country. In East Asia, married individuals tend to fear divorce because social norms regard divorced men as losers, while divorced women are likely to lose their financial sources [36,45,46]. For example, in a cross-national study using the International Social Survey Program encompassing 22 countries, the lowest rate of acceptance of divorce was found in Japan [36]. Therefore, despite their dissatisfaction with marriage, married individuals in East Asia often make painstaking efforts to maintain their marriage even if detrimental. This–a phenomenon we might call the “marriage trap”–might lead to deteriorated health in the married individuals who are unhappy with their marriages.

Lastly, satisfied marriages with facilitated interspousal communication may encourage healthy behaviors such as going to the doctor [3,47] and discourage risky behaviors such as smoking, heavy alcohol use, or illicit drug use [2,12]. By contrast, unhappy marriages may make married individuals lean towards risky behaviors. Because married men are more inclined towards unhealthy behaviors than married women, who are often more closely attached to their children, married and dissatisfied men are likely to find themselves in poorer health condition than their women counterparts. The frequency, coverage and intensity of interspousal communication may vary across countries, and the gender differences in its influences on individual health may also differ from country to country.

### Strengths and Limitations

Within the body of research studying the gender differences in the association of marriage and marital satisfaction with health, the present study has several strengths that should be highlighted. First, to our knowledge, this study was the first to analyze a dataset from a nationally representative sample survey. A few studies have previously examined this issue in Western countries [6,15,16,48,49]. However, St. John and Montgomery included very few separated and divorced people in their sample [15], and Troxel et al. [48] analyzed only a small number of women aged 42−50 years within a limited area. Holt-Lunstad et al. [49] did not distinguish divorced, widowed, and never-married individuals from single individuals. In addition, their sample was predominantly composed of white, educated, young, and healthy individuals. Lastly, in terms of methodology, the present study predicted the probability for an individual to report poor personal health, and attempted to compare different health outcomes through a cross-gender analysis by country.

The present study also had some limitations. First, although self-rated health has been widely considered useful in previous studies [50–52], objective health might represent a better measure of health than self-rated health. Second, gender differences in the inclusiveness of various types of information when reporting self-rated health might affect the gender differences in the association of marital satisfation and status with self-rated health. However, no study to date has provided a statistical method to control for these differences [53]. Third, some potential characteristics influencing self-rated health, such as smoking and drinking, could not be included as covariates as the dataset did not provide this information. Fourth, since the present study was cross-sectional in design, the definite causal relationships between individual health, marital status, and marital satisfaction could not be established. However, a recent study of 707 married individuals showed no evidence that self-rated health might affect marital status or marital stability over a 20-year period in the U.S. [5].Fifth, we used data from the 2006 EASS because the EASS dataset does not provide information about marital quality after 2006. We hope that future cross-national comparative data will include comprehensive marital quality measures. Lastly, low response rates could be another limitation of this study. However, it was reported that the samples in the 2006 EASS dataset were not statistically different from the corresponding national census data in each of the countries [28,54].

### Conclusions

A substantial body of evidence points to the salutary effects of marriage on individual health in Western countries. However, our study of four countries in East Asia suggests that depending on the gender and level of marital satisfaction, marriage can also have a negative association with individual health in certain countries. In particular, marriage dissatisfaction may lead to poor self-rated health. Besides, women may enjoy greater health benefits from marriage than men. Finally, married and dissatisfied individuals may experience a higher risk of poor health than never-married or formerly-married individuals. From a public policy perspective, further studies aimed at reducing the gender differentials in terms of health should be carried out to clarify the gender- and family-specific associations of marriage and marital satisfaction with self-rated health in other countries differing in sociocultural settings from East Asia.
